# Update on the transfusion in gastrointestinal bleeding (TRIGGER) trial: statistical analysis plan for a cluster-randomised feasibility trial

**DOI:** 10.1186/1745-6215-14-206

**Published:** 2013-07-10

**Authors:** Brennan C Kahan, Vipul Jairath, Michael F Murphy, Caroline J Doré

**Affiliations:** 1 MRC Clinical Trials Unit, Aviation House 125 Kingsway, London WC2B 6NH, UK; 2 NHS Blood & Transplant, John Radcliffe Hospital, Headley Way, Oxford OX3 9DU, UK; 3 Translational Gastroenterology Unit, Oxford, UK; 4 Transfusion Medicine, Oxford University Hospitals NHS Trust, Oxford, UK; 5 University of Oxford, Oxford, UK

**Keywords:** Statistical analysis plan, Cluster randomised trial, Transfusion, Gastrointestinal bleeding, Variceal bleeding, Feasibility trial

## Abstract

**Background:**

Previous research has suggested an association between more liberal red blood cell (RBC) transfusion and greater risk of further bleeding and mortality following acute upper gastrointestinal bleeding (AUGIB).

**Methods and design:**

The Transfusion in Gastrointestinal Bleeding (TRIGGER) trial is a pragmatic cluster-randomised feasibility trial which aims to evaluate the feasibility of implementing a restrictive *vs.* liberal RBC transfusion policy for adult patients admitted to hospital with AUGIB in the UK. This trial will help to inform the design and methodology of a phase III trial. The protocol for TRIGGER has been published in Transfusion Medicine Reviews. Recruitment began in September 2012 and was completed in March 2013. This update presents the statistical analysis plan, detailing how analysis of the TRIGGER trial will be performed. It is hoped that prospective publication of the full statistical analysis plan will increase transparency and give readers a clear overview of how TRIGGER will be analysed.

**Trial registration:**

ISRCTN85757829

## Update

### Introduction

Acute Upper Gastrointestinal Bleeding (AUGIB) is the most common reason for emergency hospital admission with a gastrointestinal disorder in the United Kingdom (UK) with an annual incidence of 50 to 150/100,000 adults [1]. It is also the leading indication for red blood cell (RBC) transfusion, accounting for 14% of all RBCs transfused in England [2]. RBC transfusion is commonly based upon the patient’s haemoglobin (Hb) level; however, the optimal Hb threshold at which to transfuse is unclear [3,4]. Two observational studies have indicated an association between RBC transfusion after AUGIB and risk of further bleeding and mortality [5,6]. A recently published randomised trial found a liberal approach to transfusion led to an increased risk of mortality [7], although these results may not be generalisable due to the strict protocols of care implemented in this single centre trial, which are unlikely to be reproducible in most healthcare institutions.

The Transfusion in Gastrointestinal Bleeding (TRIGGER) trial is a cluster-randomised trial designed to evaluate the feasibility of implementing a restrictive *vs.* liberal RBC transfusion policy for patients admitted to hospital with AUGIB [8]. TRIGGER is a pragmatic trial which aims to reflect real world settings as closely as possible. Six hospitals in the United Kingdom were randomised in a 1:1 ratio to follow either a liberal transfusion policy (where eligible patients are transfused when their Hb drops below 10 g/dL) or a restrictive transfusion policy (where eligible patients are transfused when their Hb drops below 8 g/dL). Randomisation was performed using permuted blocks without stratification or matching, with a block size of six (to ensure three hospitals in each arm). Hospitals were randomised in July 2012, and recruitment took place between September 2012 and March 2013.

The TRIGGER protocol has been published in Transfusion Medicine Reviews [8] (available at http://www.tmreviews.com), and gives details on the rationale for the study, the inclusion/exclusion criteria, the sample size calculation and the process for consenting patients to the trial. This update to the published protocol describes the statistical analysis plan for TRIGGER.

The TRIGGER trial was conducted according to the declaration of Helsinki, and received ethical approval from the Scotland A Research Ethics Committee (Reference 12/SS/0023) and the NRES Committee South Central – Oxford C (Reference 12/SC/0062).

### Outcomes

This trial will report both feasibility and clinical outcomes. Outcome definitions and explanations are available in the protocol [8].

### Feasibility outcomes

The feasibility outcomes are:

• The proportion of eligible patients who provide consent

• The proportion of screened patients who are ineligible due to need for immediate transfusion

• Overall protocol adherence

• Protocol adherence per patient

• Protocol adherence per Hb count

• Baseline characteristics of consented patients (age, shock, Hb, clinical Rockall score [9], Blatchford score [10] (two commonly used risk scores), and number of major co-morbidities for consented patients)

• The difference between consented vs non-consented patients for baseline Hb, Rockall and Blatchford scores

• The number of RBC units transfused

• The proportion of patients receiving at least one RBC transfusion

• Mean Hb over the first seven days, up to discharge, and over the entire study period

• Differences between consented patients and those discharged before being approached for consent in the Rockall and Blatchford scores, and baseline Hb.

The intra-class correlation coefficient and its 95% confidence interval from this feasibility trial will also be presented.

### Clinical outcomes

The clinical outcomes are:

• Further bleeding up to Day 28 (primary clinical outcome)

• Further bleeding up to hospital discharge

• All-cause mortality up to Day 28

• All-cause mortality up to hospital discharge

• Therapeutic intervention at the index endoscopy

• Surgical or radiological intervention to control bleeding up to death or discharge

• Occurrence of a thromboembolic or ischaemic event up to Day 28

• Occurrence of a thromboembolic or ischaemic event up to hospital discharge

• Acute transfusion reactions up to death or discharge

• Infection up to Day 28

• Infection up to hospital discharge

• Length of hospital stay

• Health related quality of life at Day 28

• Serious adverse events (SAEs) up to Day 28

The individual components that make up the composite outcome of a thromboembolic or ischaemic event (myocardial infarction, stroke, pulmonary embolus, deep vein thrombosis, and acute kidney injury) will also be analysed separately at both Day 28 and at hospital discharge.

## Analysis principles

### Feasibility outcomes

Analysis of the feasibility outcomes will be by intention-to-treat (ITT), and will include all consented patients on whom an outcome is available, unless otherwise stated. A secondary analysis will include all consented patients with a recorded Hb below 12 g/dL during follow-up, and for whom an outcome is available (this secondary analysis will be restricted to RBC transfusion outcomes, Hb concentration outcomes, and adherence outcomes). A 5% significance level will be used. All analyses of feasibility outcomes will be unadjusted for baseline covariates.

### Clinical outcomes

Main analysis of the clinical outcomes will be by ITT, and will include all consented patients with a recorded Hb below 12 g/dL during follow-up, and for whom the outcome is available. Including only patients with a Hb <12 g/dL allows us to target those patients most likely to be affected by the treatment policy, resulting in a more powerful analysis on a more relevant patient population. A Hb of 12 g/dL was chosen as the cut-off point because it is likely that some patients will be transfused (against policy) above 10 g/dL; if the proportion of patients transfused above 10 g/dL differs between treatment arms, excluding these patients could lead to bias. Using a cut-off of 12 g/dL should allow the majority of transfused patients to be included in the analysis, leading to an unbiased comparison. A secondary analysis will include all consented patients, regardless of whether their Hb dropped below 12 g/dL.

Results will be considered statistically significant at the 5% level. Main analyses for clinical outcomes will be unadjusted for baseline covariates; however, a set of secondary analyses will be adjusted for patient age, the presence of shock, the number of major co-morbidities, and the presence of coagulopathy (defined as an international normalised ratio (INR) >1.5 or a prothrombin time (PT) >3 seconds greater than the control). Mean imputation within the centre will be used for patients with missing baseline covariates [11]. Patient age and the number of major co-morbidities (encompassing ischaemic heart disease, cardiac failure, liver disease, renal disease, respiratory disease, malignancy and stroke) will be modelled using fractional polynomials to allow for the possibility of a non-linear association [12]. Further bleeding up to Day 28 is regarded as the primary clinical outcome.

### Analysis methods

All analyses will account for clustering to ensure correct type I error rates and confidence intervals [13-15]. Many cluster randomised trials base their analysis on individual level patient data, and use appropriate statistical methods to account for clustering between patients in the same cluster (for example, mixed-effects models or generalised estimating equations). However, analysis methods based on individual level patient data may not perform well when the number of clusters is small [13,14]. Analysis for TRIGGER will, therefore, be performed using cluster-level summaries, which performs well even with a very small number of clusters [13,14].

Equal weight will be given to each of the six clusters. All analyses will compare the two treatment arms, unless otherwise stated. Binary outcomes will be presented as a difference in proportions.

### Unadjusted analyses

Unadjusted analyses using cluster-level summaries can be performed by calculating a summary outcome from each centre, and fitting a linear regression model with the summary outcome as a response variable, and treatment arm as a covariate. For example, for the outcome of mortality, one might choose the proportion of patients who died as a summary measure. To perform the analysis, one would then need to calculate the proportion of patients who died in each centre. A linear regression model would then be fitted, with the proportion of patients who died in each centre as the outcome, and which treatment the centre was randomised to as a covariate (in the TRIGGER trial there would only be six data points, as there are only six centres).

### Adjusted analyses

Adjusted analyses using cluster-level summaries will be performed as follows [14]:

1) A regression model (linear for continuous outcomes and logistic for binary outcomes) will be fit to individual-level patient data, and will be adjusted for the baseline characteristics listed earlier (age, shock, presence of coagulopathy and the number of major co-morbidities). The model will not adjust for treatment effect, or for centre.

2) Predicted values based on the fitted regression model will be calculated for each patient (for binary outcomes this equates to the predicted probability of experiencing an event, for continuous outcomes this equates to the predicted mean).

3) The expected outcome in each cluster will be calculated. For binary outcomes, this is the expected number of events in each cluster, and is calculated by summing the predicted probabilities for each patient in that cluster. For continuous outcomes, the expected mean value is calculated by taking the mean of the predicted values in each cluster.

4) An appropriate residual is calculated for each cluster. For binary outcomes, this is the observed number of events minus the expected number of events, divided by the number of patients in the cluster. For continuous outcomes, this is the observed mean minus the expected mean.

5) A linear regression model will be fit using the residuals calculated above as cluster-level summaries, with only the treatment group as a covariate. No degrees of freedom correction will be made for performing an adjusted analysis, as only patient-level variables will be adjusted for.

### Cluster level summaries for feasibility outcomes

Cluster-level summaries for feasibility outcomes will be calculated separately in each centre as follows:

1) **Recruitment rate (patients consenting):** the proportion of eligible patients providing consent.

2) **Ineligible due to severity of bleeding:** the proportion of screened patients who are ineligible due to severity of bleeding

3) **Overall adherence:** the mean adherence rate per patient

4) **Adherence per patient:** the proportion of patients who had no protocol deviations

5) **Adherence per Hb count:** the proportion of Hb counts that did not lead to a protocol deviation

6) **Selection bias - baseline variables for consented patients:** The mean age, baseline Hb, number of major co-morbidities, clinical Rockall score and Blatchford score, and the proportion of patients with shock will be calculated for consented patients.

7) **Selection bias - difference between consented and non-consented patients in baseline variables:** The difference in the mean Rockall score and Blatchford score, and mean baseline Hb between consented and non-consented patients will be calculated in each centre.

8) **Red blood cell exposure (number of transfusions):** the mean number of RBC units transfused per patient.

9) **Red blood cell exposure (patients receiving at least one transfusion):** the proportion of patients who receive at least one RBC transfusion.

10) **Hb concentration up to Day 7, and over the entire in-hospital follow-up period, prior to discharge/death/Day28:** the area-under-the-curve will be calculated for each patient

11) **Hb concentration at discharge:** the mean Hb will be calculated

### Cluster level summaries for clinical outcomes

The cluster-level summary will be calculated as the proportion of patients in each centre experiencing the event of interest for the following outcomes: further bleeding, all-cause mortality, need for therapeutic intervention at index endoscopy, need for surgery or radiological intervention to control bleeding, any thromboembolic or ischaemic events (and each of the components separately), acute transfusion reactions, infections and SAEs.

A cluster-level summary for length of hospital stay will be calculated using the median length of stay in each centre, and a cluster-level summary for health related quality of life will be calculated using the mean EQ-5D [16] in each centre.

### Subgroup analyses

Subgroup analyses will be performed for two outcomes: further bleeding and all-cause mortality (both up to Day 28) using an interaction test, and considered statistically significant at the 5% level. The following subgroup analyses will be performed:

• Variceal vs. non-variceal bleeding

• Ischaemic heart disease vs. no ischaemic heart disease

Interaction tests will be performed by calculating the difference in proportions (for the chosen outcome) between subgroups within each centre [14]. A linear regression model will then be fit, with the difference in proportions between subgroups as the outcome, and treatment as a covariate. Interaction tests will be unadjusted for baseline covariates, and will be reported with a 95% confidence interval.

### Sensitivity analyses

Missing data for each clinical outcome will be summarised by treatment arm. Sensitivity to missing data for further bleeding and all-cause mortality up to Day 28 will be assessed under a range of missing-not-at-random scenarios. This will be done by calculating the mean outcome in each cluster as follows:

$$\left(x_{\mathrm{obs}}\right)\left(1-p_{\mathrm{missing}}\right)+\left(x_{\mathrm{sensitivity}}\right)\left(p_{\mathrm{missing}}\right)$$

where *x*_*obs*_ is the observed cluster level summary, *p*_*missing*_ is the proportion of patients with missing data in that cluster, and *x*_*sensitivity*_ is the proportion of patients with missing data who are assumed to have had the event of interest. *x*_*sensitivity*_ will be varied between 0, 0.2, 0.4, 0.6, 0.8 and 1. The treatment effect and 95% confidence interval will be calculated as before.

## Discussion

It has become increasingly common in recent years to prospectively publish trial protocols to increase transparency by allowing a comparison between what was presented in the final manuscript and what was planned in the protocol before trial results were known [17-20]. The rationale for prospectively publishing trial protocols also applies to statistical analysis plans.

There are generally a number of different ways to analyse an outcome in a clinical trial. For example, analyses could adjust for different covariates, or be completely unadjusted [21-24]; if covariates were adjusted for in the analysis, this could be performed in different ways (for example, using Mantel-Haenszel estimates, or an adjusted logistic regression model for binary outcomes [25]). In multicentre trials, one could either ignore the centre-effects, or account for them in a number of different ways (for example, using fixed or random effects [26-28]). Issues regarding multiplicity (for example, when taking multiple looks at the data through interim analyses, or through multiple pairwise comparisons between treatment groups) may or may not be accounted for in the analysis [29].

It is hoped that results will be robust, and will give similar conclusions regardless of the method of analysis used. However, different analysis methods will often give different results, either through chance or because some analysis methods are more appropriate in certain situations. It is important for trialists to pre-specify the method of analysis to prevent them from assessing the data using several different methods of analysis, and presenting the most favourable result.

The benefits from a prospectively published statistical analysis plan can be seen from a randomised trial published in 2013 [30]. The trial used a group sequential design, which is known to lead to biased estimates of treatment effect and inflated type I error rates if it is not properly accounted for in the trial analysis [29]. In this trial, accounting for the group sequential nature of the trial would have given a non-significant result for the primary outcome, whereas ignoring it gave a statistically significant result. The manuscript presented the significant result as their primary analysis; had the statistical analysis plan been prospectively published prior to data analysis it would have given readers more confidence that the method of analysis had not been chosen based on whether or not it gave a statistically significant result.

We are, therefore, prospectively publishing the statistical analysis plan for the TRIGGER trial to increase transparency, and to provide readers with a more complete picture of the methods that will be used to analyse the trial. The statistical analysis plan was signed off in February 2013, before any trial investigators had access to the trial database. Recruitment to TRIGGER began in September 2012 and finished in March 2013.

## Abbreviations

AUGIB: Acute upper gastrointestinal bleeding; Hb: Haemoglobin; INR: International normalised ratio; ITT: Intention-to-treat; PT: Prothrombin time; RBC: Red blood cell; SAE: Serious adverse event; TRIGGER: Transfusion in gastrointestinal bleeding trial.

## Competing interests

The authors declare that they have no competing interests.

## Authors’ contributions

BK wrote the initial draft of the manuscript. VJ, CD and MM provided important input into the manuscript. All authors approved the final version of the manuscript.

## List of recruiting centres

Edinburgh Royal Infirmary - Dr Alasdair Gray, Dr Nick Church

Glasgow Royal Infirmary - Dr Adrian Stanley

St James Hospital Leeds - Dr Simon Everett

James Cook Hospital Middlesbrough - Dr Helen Dallal, Dr John Greenaway

Queens Medical Centre Nottingham - Dr Martin James, Dr Ivan Le Jeune

Oxford University Hospital - Dr Adam Bailey, Dr Melanie Darwent and Dr Ian Reckless
